# Severe Polypoidal Choroidal Vasculopathy as a Harbinger of Spontaneous Suprachoroidal Hemorrhage

**DOI:** 10.1177/24741264261442930

**Published:** 2026-04-21

**Authors:** Brendan K. Tao, Austin Pereira, Mohamed Gemae, Nupura Bakshi, Tina Tang, Panos Christakis, Miguel Cruz-Pimentel, Peng Yan

**Affiliations:** 1 Department of Ophthalmology and Vision Sciences, University of Toronto, Ontario, Canada; 2 Dean McGee Eye Institute, Department of Ophthalmology, The University of Oklahoma, USA

**Keywords:** polypoidal choroidal vasculopathy, submacular hemorrhage, subretinal hemorrhage, suprachoroidal hemorrhage, spontaneous

## Abstract

**Purpose:** To report a rare case of spontaneous suprachoroidal hemorrhage in polypoidal choroidal vasculopathy (PCV) and propose a possible pathophysiologic cascade with associated clinical warning signs. **Methods:** A single observational case was reviewed. **Results:** A 66-year-old man with PCV developed sequential massive submacular hemorrhage, breakthrough vitreous hemorrhage, non-appositional choroidal elevation, and ultimately appositional suprachoroidal hemorrhage with angle closure and ocular hypertension. Despite treatment with posterior sub-Tenon triamcinolone and conservative management, the final visual outcome was no light perception. **Conclusions:** Spontaneous suprachoroidal hemorrhage in PCV is an extremely rare but devastating event. Based on the patient’s stepwise clinical progression over several follow-up visits, we propose a sentinel cascade involving severe choroidal and vortex vein congestion culminating in blowout hemorrhage into the suprachoroidal space. Recognition of red-flag features may aid in early identification and closer surveillance of high-risk cases.

## Introduction

Polypoidal choroidal vasculopathy (PCV), a distinct phenotype of neovascular age-related macular degeneration (AMD), is characterized by aneurysmal dilations of the inner choroidal vasculature and an associated branching vascular network.^1^ PCV frequently presents with recurrent serosanguinous pigment epithelial detachments (PEDs) and subretinal vascular lesions, often accompanied by hemorrhage, which may ultimately lead to visual loss.^1^ Reported complications include exudative retinal detachment, chorioretinal atrophy, fibrotic scarring, and, in extremely rare cases, suprachoroidal hemorrhage.^2^

Although suprachoroidal hemorrhage is frequently associated with intraocular surgery or trauma,^3^ it may rarely occur spontaneously in cases associated with markedly fragile choroidal vasculature. Spontaneous suprachoroidal hemorrhage secondary to PCV was first described by Tan et al,^2^ who reported a case initially presenting with exudative retinal detachment in newly diagnosed PCV. Over 5 days, the patient experienced rapid deterioration, culminating in suprachoroidal hemorrhage and secondary angle closure.^2^ Herein, we describe a distinct case of progressive, stepwise spontaneous suprachoroidal hemorrhage with secondary angle closure, which developed over 3 weeks in an eye with long-standing PCV and submacular hemorrhage (SMH).

## Case Report

A 66-year-old man with a history of diabetes, hypertension, and dyslipidemia was referred for sudden vision loss in the left eye secondary to a new large SMH. His ocular history was significant for PCV, which had been observed for approximately 20 years. In the left eye, this had progressed to a massive, painless subretinal pigment epithelium (RPE) hemorrhage 2 years before the current presentation. At that time, the hemorrhage involved the macula, extending inferiorly to the equator, and was associated with both subretinal and vitreous hemorrhage (VH).

**Figure 1.**
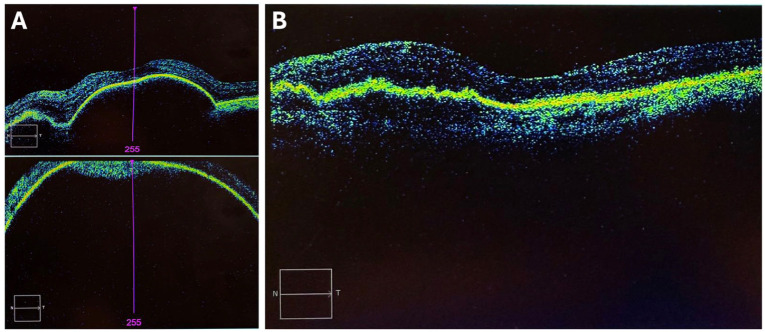
(A) Spectral-domain optical coherence tomography (OCT) of the left eye obtained 2 years before the current presentation demonstrating a massive retinal pigment epithelium (RPE) detachment centrally (top panel) and extending inferiorly, with elevation approaching the limits of the imaging field (bottom panel). This occurred in the setting of the patient’s first massive sub-RPE hemorrhage, which was painless and involved the macula, extended inferiorly to the equator, and was associated with subretinal and vitreous hemorrhage. (B) Repeat OCT obtained 2 years after the initial hemorrhage demonstrating a stable, low-lying RPE detachment. This image was taken 6 weeks before the current presentation with a second massive submacular hemorrhage, which subsequently progressed to spontaneous suprachoroidal hemorrhage in the setting of polypoidal choroidal vasculopathy.

Spectral-domain optical coherence tomography (SD-OCT) demonstrated a massive RPE detachment extending inferiorly, with elevation approaching the limits of the imaging field (Figure 1A). The patient was subsequently managed with intravitreal (IVT) antivascular endothelial growth factor (anti-VEGF) aflibercept injections for the next 2 years, with treatment intervals extended to 8 weeks. His best-corrected distance visual acuity (BCVA) improved from counting fingers to 20/40, as recorded 6 weeks before his current presentation. OCT at that time showed a stable, low-lying RPE detachment (Figure 1B). A review of his previous ophthalmologic records revealed no atypical features of PCV, with only a single localized peripapillary polyp of unremarkable size.

**Figure 2.**
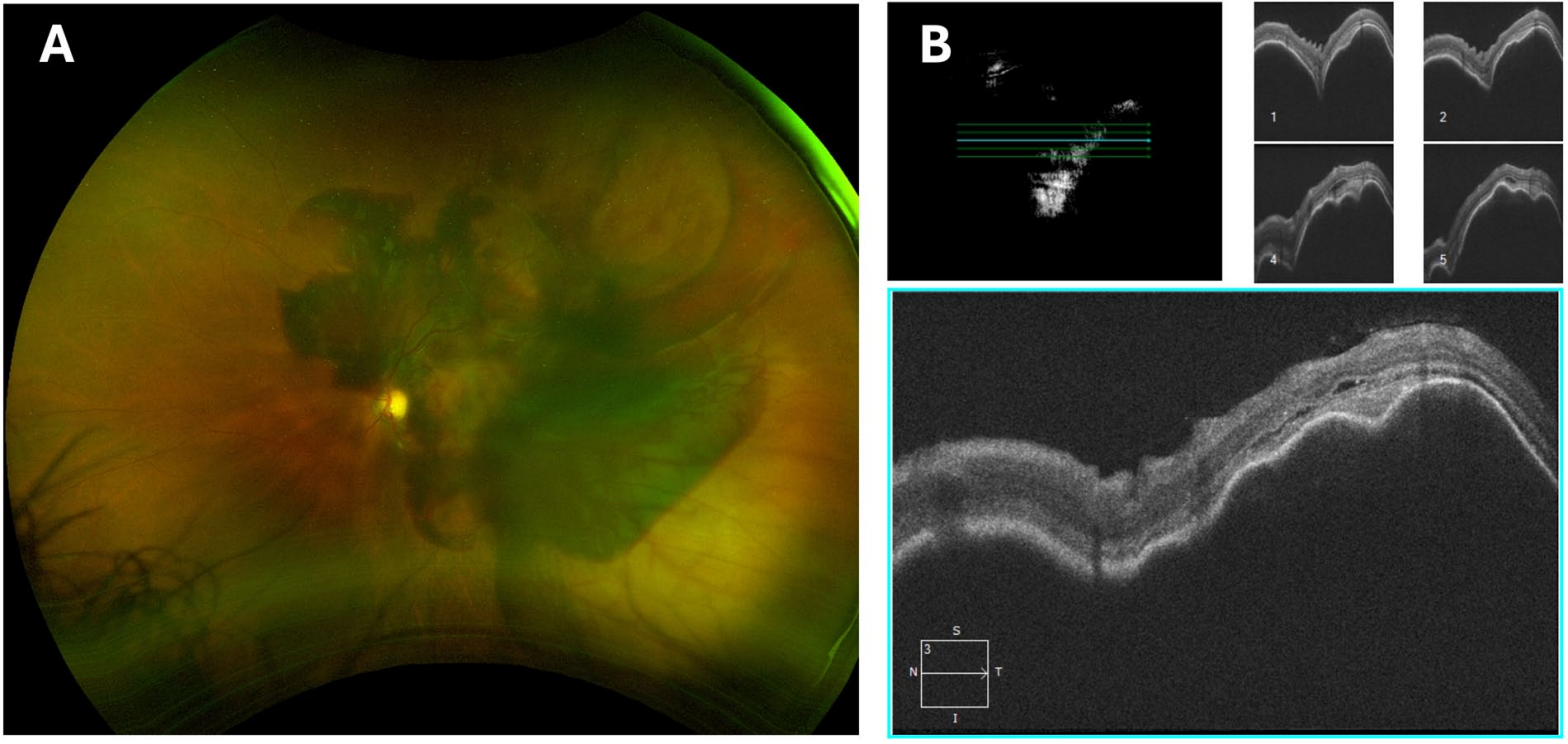
(A) Color fundus photography of the left eye at initial presentation demonstrating a massive submacular hemorrhage (SMH) in the posterior pole and extending beyond the superior and inferior vascular arcades, with visible overlying retinal vasculature. (B) Spectral-domain optical coherence tomography 5-line raster of the left eye at initial presentation showing diffuse SMH involving both the subretinal and subretinal pigment epithelial spaces, with multiple pigment epithelial detachments.

At the current presentation, 6 weeks after the patient’s last IVT injection, his BCVA was 20/20 OD and hand motions (HM) OS. Intraocular pressures were 14 mm Hg OD and 10 mm Hg OS. Anterior segment examination was unremarkable. Fundus examination of the left eye revealed a large, diffuse SMH involving both the subretinal and sub-RPE spaces, with multiple PEDs (Figure 2A), while the right macula was unremarkable. SD-OCT confirmed the extent of the hemorrhagic PED (Figure 2B).

**Figure 3.**
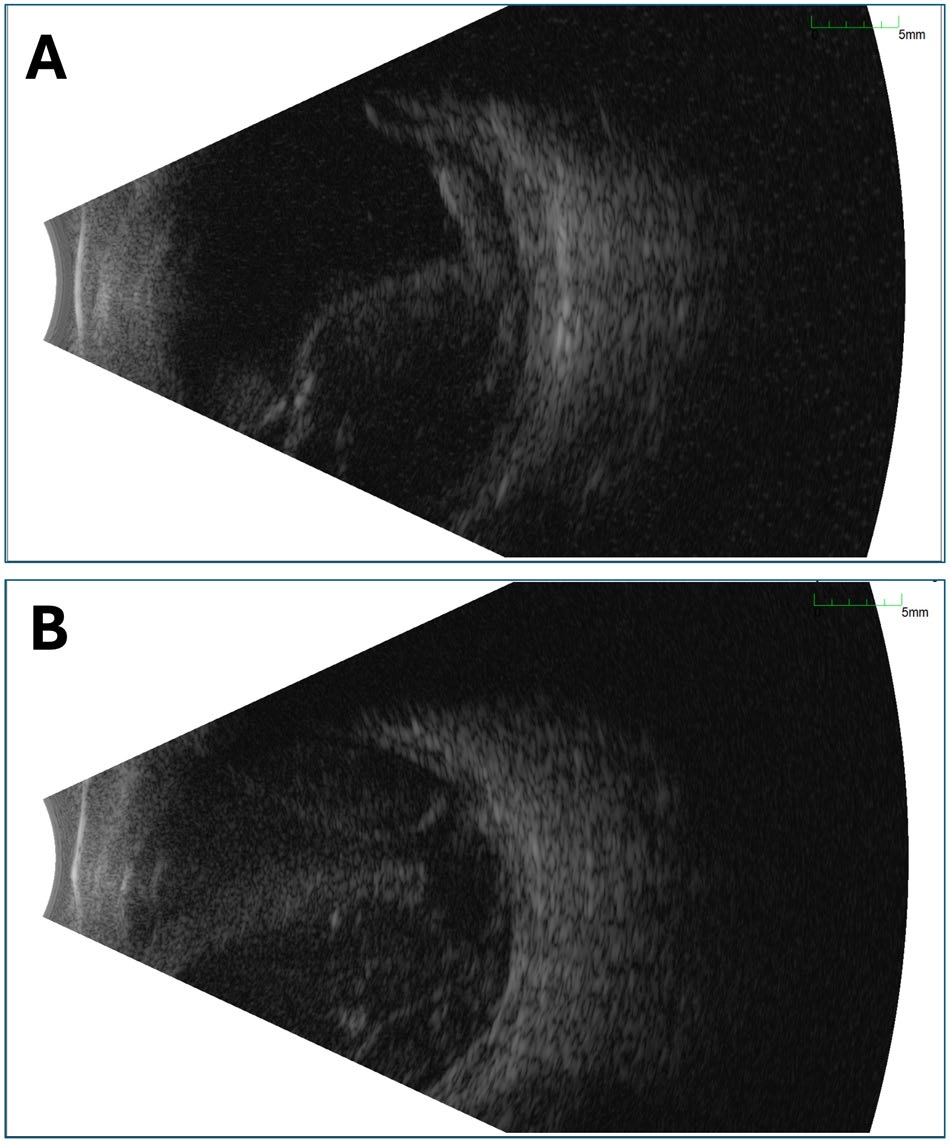
(A) B-scan ultrasonography of the left eye at 1-week follow-up demonstrating smooth, dome-shaped elevations arising from the peripheral walls of the globe, consistent with a large, non-appositional choroidal detachment of hemorrhagic origin. A mobile, medium-to-low reflectivity echo is also present within the vitreous cavity, consistent with breakthrough vitreous hemorrhage. (B) B-scan ultrasonography of the left eye at 3-week urgent presentation for severe ocular pain and no light perception vision demonstrating a massive, appositional suprachoroidal hemorrhage occupying the majority of the vitreous cavity and causing anterior displacement of the iris–lens diaphragm.

Given the poor candidacy for surgical intervention with subretinal tissue plasminogen activator (tPA) displacement, the patient elected for observation. A week later, he returned with persistent HM vision in the left eye, with an intraocular pressure of 9 mm Hg. A new breakthrough VH was noted, and B-scan ultrasonography revealed a large, non-appositional choroidal elevation consistent with hemorrhagic choroidal detachment (Figure 3A). Surgical options, including pars plana vitrectomy (PPV) with choroidal drainage, were discussed; however, given the guarded visual prognosis, the patient elected to undergo a trial of posterior sub-Tenon triamcinolone acetonide injection to reduce choroidal edema.^4^

Two weeks thereafter, the patient presented to the emergency department with severe pain in the left eye and no light perception vision. He denied any preceding Valsalva maneuver or trauma. Intraocular pressure was 50 to 60 mm Hg, with a shallow anterior chamber centrally and peripherally, accompanied by diffuse corneal epithelial edema. B-scan ultrasonography demonstrated an appositional suprachoroidal hemorrhage with forward displacement of the lens–iris diaphragm, resulting in a secondary angle-closure attack due to a posterior pushing mechanism (Figure 3B). Given the poor visual potential, the patient was managed conservatively with oral acetazolamide and topical aqueous suppressants, along with gabapentin and opioids for analgesia. After 24 hours, his pain was well controlled, and intraocular pressure improved to 20 mm Hg OS. Enucleation or evisceration was discussed in the event of intractable pain. At the 3-month follow-up, vision remained at no light perception; however, pain had resolved following cyclophotocoagulation, with intraocular pressure measuring 24 mm Hg.

## Conclusions

Suprachoroidal hemorrhage remains one of the most feared complications in ophthalmology owing to its sudden onset, profound vision loss, and limited therapeutic options. While it is classically associated with intraocular surgery or trauma, it may also occur in eyes with extreme vascular fragility, such as those affected by PCV. To date, only a few cases of spontaneous suprachoroidal hemorrhage in PCV have been reported in the literature, typically characterized by abrupt onset and catastrophic visual outcomes.

Spontaneous suprachoroidal hemorrhage is associated with several systemic and ocular risk factors. Systemic factors include advanced age, anticoagulant or thrombolytic therapy, systemic hypertension, atherosclerosis, diabetes mellitus, blood dyscrasias, chronic renal disease, and Valsalva maneuver.^5^ Beyond PCV, other ocular factors are AMD, glaucoma, and high myopia.^5^ Treatment involves medication to reduce intraocular pressure, laser iridotomy for angle closure if present, and surgery with drainage via sclerotomy or vitrectomy. In cases of uncontrolled pain or recurrent hemorrhage, evisceration or enucleation may be required.^5^ Visual prognosis remains poor.^5^ In a case report and systematic review of suprachoroidal hemorrhage in patients with AMD, Hsiao et al^5^ reported presenting visual acuity of HM or worse in 71.4% of patients, with final visual acuity of HM or worse in 80%, and no light perception in 63.3%. Table 1 compares the clinical course of our patient with that of the patient reported by Tan et al.^2^

**Table 1.** Review of Published Cases of Polypoidal Choroidal Vasculopathy-Associated Spontaneous Suprachoroidal Hemorrhage.

Case	Patient demographics	Risk factors	Presentation	Progression	Management	Outcome
Tan et al, 2007	61-year-old woman, no systemic comorbidities reported; newly diagnosed PCV	Spontaneous; no trauma, Valsalva maneuver, or anticoagulation	Painless vision loss in the right eye; extensive exudative retinal detachment with subretinal hemorrhage and pulsatile choroidal polyps	Rapid progression to massive appositional SCH with secondary angle closure over 5 days, with recurrent SCH by 2 weeks	Conservative: intraocular pressure-lowering medications, laser peripheral iridotomy, and iridoplasty	No light perception; phthisical, asymptomatic eye
Current case	66-year-old man with DM, hypertension, and dyslipidemia; longstanding PCV with prior anti-VEGF therapy	Spontaneous; no trauma, Valsalva maneuver, or anticoagulation	Sudden vision loss with massive submacular hemorrhage	Stepwise progression over 3 weeks	Conservative: intraocular pressure-lowering medications, analgesia, and posterior sub-Tenon corticosteroid injection	No light perception; pain controlled; eventual ocular palliation

Abbreviations: anti-VEGF, antivascular endothelial growth factor; DM, diabetes mellitus; F, female; M, male; PCV, polypoidal choroidal vasculopathy; SCH, suprachoroidal hemorrhage.

We present a rare case of spontaneous suprachoroidal hemorrhage occurring in the absence of preceding Valsalva maneuver, anticoagulant therapy, trauma, or intraocular surgery, in a vasculopathic patient with longstanding PCV and extensive SMH. While a prior case proposed a mechanism involving shearing of fragile blood vessels as the vasculature enters the suprachoroidal space,^2^ our case is distinct in demonstrating a sentinel, stepwise progression from massive SMH to VH, non-appositional choroidal elevation, and ultimately appositional suprachoroidal hemorrhage with secondary angle closure over a 3-week period.

**Figure 4.**
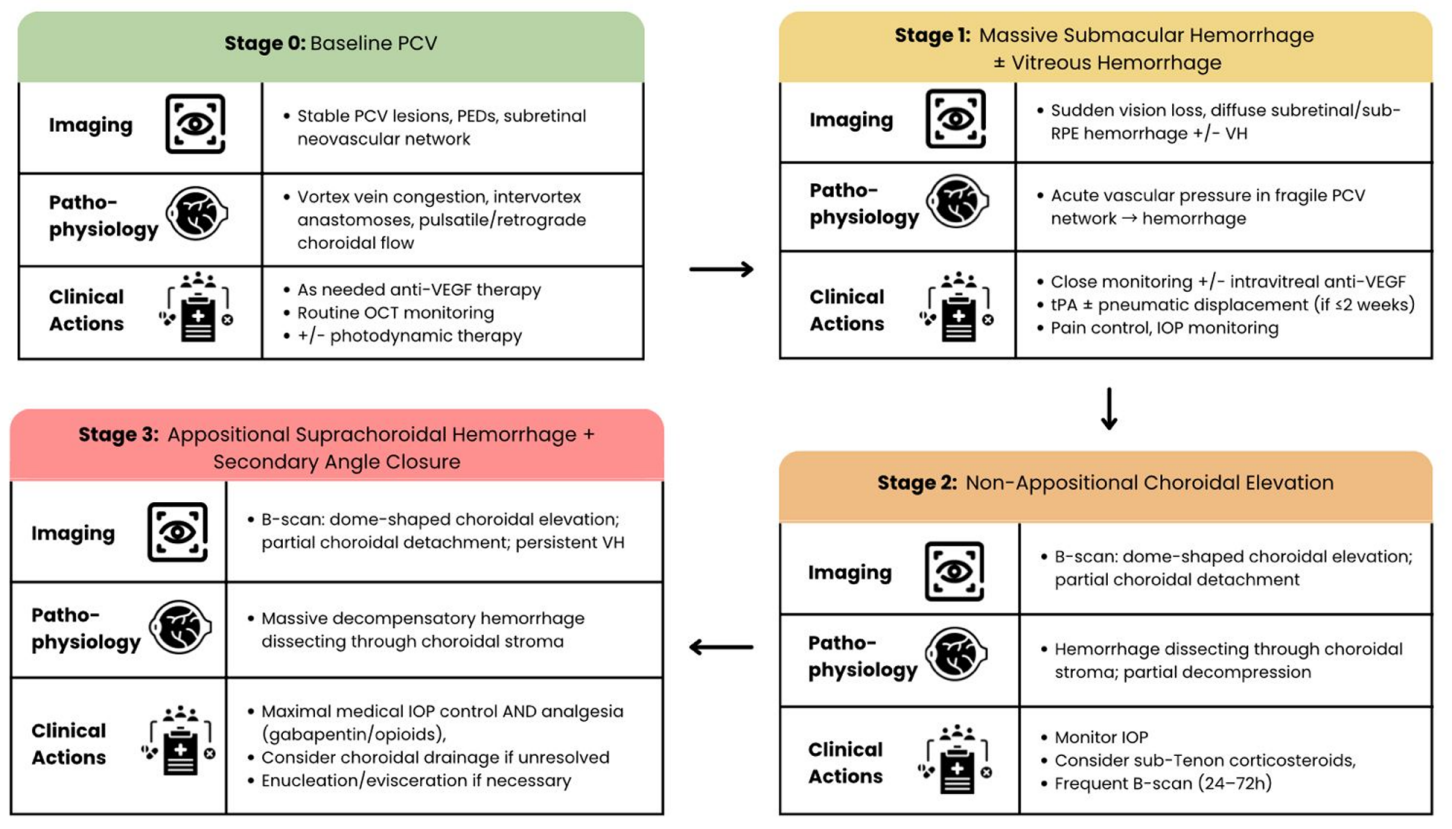
Proposed sentinel cascade of suprachoroidal hemorrhage in severe polypoidal choroidal vasculopathy, illustrating stepwise progression from baseline disease to massive submacular hemorrhage, subsequent vitreous hemorrhage, non-appositional choroidal elevation, and ultimately appositional suprachoroidal hemorrhage with secondary angle closure.

Based on our patient’s clinical course, we propose a 4-stage cascade of PCV-associated suprachoroidal hemorrhage (Figure 4): i) stage 0: baseline PCV characterized by pachyvessels, vortex vein congestion, and recurrent serosanguinous PEDs; ii) stage 1: massive SMH with or without breakthrough VH, reflecting a sudden rise in choroidal vascular pressure; iii) stage 2: non-appositional choroidal elevation, representing progressive dissection of blood into the suprachoroidal space without angle compromise; and iv) stage 3: appositional suprachoroidal hemorrhage with forward displacement of the lens–iris diaphragm and secondary angle closure. This framework contrasts with postoperative suprachoroidal hemorrhage, in which hypotony and ciliary body traction are the predominant mechanisms.^3^ In PCV, however, the mechanism may involve severe choroidal congestion, intervortex anastomoses, pachyvessel fragility, and impaired vortex vein outflow, compounded by systemic vasculopathy (eg, hypertension and diabetes). Such pathophysiology may account for both the predisposition to recurrent massive SMH and the eventual blowout of blood into the suprachoroidal space.

Table 2 summarizes the clinical implications of spontaneous suprachoroidal hemorrhage in PCV, including red flag features, surveillance recommendations, and management considerations. In cases of massive SMH with new VH and choroidal elevation, we recommend close surveillance with sequential B-scan ultrasonography and intraocular pressure monitoring every 24 to 72 hours. Treatment options include IVT anti-VEGF therapy, IVT tPA with or without pneumatic displacement, and PPV with subretinal tPA.^6^ Early pain management may help reduce elevations in systemic blood pressure, which may otherwise increase the risk of suprachoroidal hemorrhage. In cases of significant suprachoroidal hemorrhage that do not respond to medical management or when severe ocular pain continues, choroidal drainage could be considered. Other surgical indications include uncontrolled intraocular pressure, appositional choroidal detachment, retinal detachment, and macular involvement.^7^

**Table 2.** Summary of Clinical Implications, Including Red Flag Features, Proposed 4-Stage Cascade, Surveillance Recommendations, and Management Considerations, for Spontaneous Suprachoroidal Hemorrhage in Polypoidal Choroidal Vasculopathy.

Red Flag Features (High Risk for SCH in PCV)	Pathophysiology: Proposed 4-Stage Cascade	Surveillance Recommendations	Management Considerations
Vasculopathic risk factors in a patient with PCV	Stage 0: Baseline PCV with pachyvessels and recurrent serosanguinous PEDs	Routine follow-up	Intravitreal anti-VEGF therapy for underlying PCV activity
Massive SMH, especially if ≥3-disc diameters; breakthrough vitreous hemorrhage following SMH	Stage 1: Massive SMH ± breakthrough vitreous hemorrhage	After large SMH with new VH or choroidal elevation, consider B-scan ultrasonography and intraocular pressure monitoring every 24–72 hours	Surgical options: i) Intravitreal tPA + pneumatic displacement or PPV + subretinal tPA within 14 days if visual potential exists
Non-appositional choroidal elevation on B-scan ultrasonography (smooth dome-shaped detachment)	Stage 2: Non-appositional choroidal elevation	Continue close monitoring with serial B-scan ultrasonography and intraocular pressure assessment every 24 hours	Medical therapy: Pain control, intraocular pressure-lowering agents, cautious systemic BP management Surgical options: Choroidal drainage if appositional SCH persists with severe pain or uncontrolled intraocular pressure
Rising intraocular pressure or early shallowing of the anterior chamber with appositional SCH.	Stage 3: Appositional SCH with angle closure	Prognosis: Visual recovery is poor once appositional SCH and angle closure occur; early recognition is key	Pain control, intraocular pressure management, palliative ocular management

Abbreviations: BP, blood pressure; PED, pigment epithelial detachment; PCV, polypoidal choroidal vasculopathy; SCH, suprachoroidal hemorrhage; SMH, submacular hemorrhage; tPA, tissue plasminogen activator.

Finally, our case highlights the limitations of conservative and nonsurgical therapy. Despite posterior sub-Tenon triamcinolone administered to reduce inflammation and vascular permeability,^4,8^ the patient still progressed to suprachoroidal hemorrhage and ultimately required ocular palliation.

In conclusion, we propose the first staged sentinel cascade of suprachoroidal hemorrhage in PCV, which may improve early recognition and management of this rare but devastating complication. This case highlights a stepwise progression from massive SMH to VH, non-appositional choroidal elevation, and ultimately appositional suprachoroidal hemorrhage with angle closure. Recognition of intermediate stages—particularly massive SMH with new VH and evolving choroidal elevation—should prompt close surveillance with serial B-scan ultrasonography and intraocular pressure monitoring every 24 to 72 hours. Key red-flag features include large SMH with breakthrough VH, progressive dome-shaped choroidal elevation, and rising intraocular pressure or early shallowing of the anterior chamber.

We recommend a risk-stratified monitoring strategy for patients with severe PCV and SMH, balancing early intervention (eg, IVT tPA with pneumatic displacement or vitrectomy) against the high risk of surgical complications in eyes with poor visual potential. By integrating a detailed clinical course with a proposed staged framework, this report provides a practical approach for anticipating disease progression. Future multicenter studies are needed to validate this cascade and identify predictive markers of progression. Ultimately, early recognition of warning stages may enable timely intervention before irreversible appositional suprachoroidal hemorrhage and secondary angle closure occur.
